# Kinesin-14 and kinesin-5 antagonistically regulate microtubule nucleation by γ-TuRC in yeast and human cells

**DOI:** 10.1038/ncomms6339

**Published:** 2014-10-28

**Authors:** Zachary T. Olmsted, Andrew G. Colliver, Timothy D. Riehlman, Janet L. Paluh

**Affiliations:** 1 State University of New York Polytechnic Institute, College of Nanoscale Science, Nanobioscience Constellation, Albany, New York 12203, USA

## Abstract

Bipolar spindle assembly is a critical control point for initiation of mitosis through nucleation and organization of spindle microtubules and is regulated by kinesin-like proteins. In fission yeast, the kinesin-14 Pkl1 binds the γ-tubulin ring complex (γ-TuRC) microtubule-organizing centre at spindle poles and can alter its structure and function. Here we show that kinesin-14 blocks microtubule nucleation in yeast and reveal that this inhibition is countered by the kinesin-5 protein, Cut7. Furthermore, we demonstrate that Cut7 binding to γ-TuRC and the Cut7 BimC domain are both required for inhibition of Pkl1. We also demonstrate that a yeast kinesin-14 peptide blocks microtubule nucleation in two human breast cancer cell lines, suggesting that this mechanism is evolutionarily conserved. In conclusion, using genetic, biochemical and cell biology approaches we uncover antagonistic control of microtubule nucleation at γ-TuRC by two kinesin-like proteins, which may represent an attractive anti-mitotic target for cancer therapies.

## Abstract (web summary)

Mitotic spindle assembly requires strict control of microtubule nucleation by γ-tubulin ring complexes. Olmsted *et al*. report that the kinesin-like proteins Pkl1 and Cut7 antagonistically regulate nucleation in fission yeast, and show that a Pkl1 peptide blocks spindle assembly in human cancer cells.

## Graphical abstract

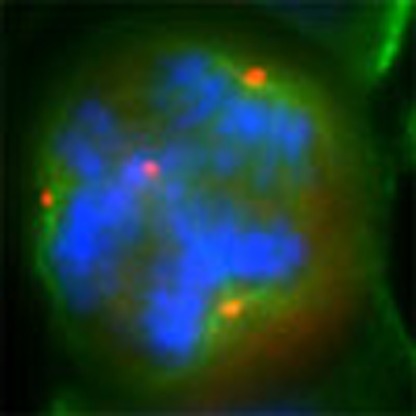

The microtubule cytoskeleton is a self-assembling network that underlies specialized, often polarized, cellular functions in eukaryotes. Knowledge of its mechanisms is fundamental to understanding normal development and disease and is expected to assist new technologies through biomimicry. The microtubule-based mitotic spindle apparatus is perhaps the best studied self-assembly platform^1^,^2^ and a primary target for cancer therapeutics^3^. Spindle pole microtubule-organizing centres (MTOCs) utilize a γ-tubulin template within a ring complex (γ-tubulin ring complex, γ-TuRC) to orchestrate addition of α-/β-tubulin heterodimeric microtubule building blocks into 25 nm polarized microtubules^4^,^5^,^6^,^7^,^8^,^9^. Conserved protein structural features of the γ-TuRC MTOC have been identified through crystallography studies from multiple model organisms and include α-/β-tubulin^10^, γ-tubulin^11^, GCP4 (ref. 12) and the γ-tubulin small complex (γ-TuSC) cryo-EM structure^13^. Conserved structural features are additionally supported by cross-species analysis^14^,^15^. Still unknown is how dynamic control over MTOC functions for microtubule nucleation and organization is achieved. The fission yeast *Schizosaccharomyces pombe* provides an ideal eukaryotic platform to address conserved MTOC mechanisms^14^,^15^,^16^,^17^.

The coordination of spindle microtubules into a bipolar array requires kinesin-like proteins (Klps), though Klp mitotic functions are not limited to interactions solely on microtubules. Studies of the functionally diverse kinesin-14 Klp family across eukaryotes have indicated an ability by some members to affect microtubule number and organization at spindle poles^18^,^19^,^20^,^21^. In fission yeast, kinesin-14 Pkl1 interacts directly with the γ-TuRC MTOC to alter its composition and function^17^,^22^,^23^. Conservation of the kinesin-14 γ-TuRC regulatory mechanism is expected from yeast to human, as human kinesin-14 HSET replaces fission yeast kinesin-14 Pkl1 (ref. 23) and all human γ-TuSC protein components are also compatible^14^,^15^. Nearly as ubiquitous and complex in eukaryotes as kinesin-14 Klps are members of the kinesin-5 family that oppose kinesin-14 function. In fission yeast, kinesin-5 Cut7 opposes the action of kinesin-14 Pkl1 in mitosis, but the detailed mechanism is not yet characterized. Elucidating this mechanism could be informative for understanding γ-TuRC regulation and spindle bipolarity.

In this study, we expand the mechanism for kinesin-14 regulation of γ-TuRC. Studies from our lab and others describe genetic interactions of Pkl1 with γ-TuRC proteins^22^,^24^,^25^,^26^, checkpoint pathways^20^,^26^ and spindle pole organization^20^. More recently we identified key Tail elements in Pkl1 that function along with Motor binding to γ-tubulin to regulate γ-TuRC^17^,^22^,^23^. Here we demonstrate that kinesin-14 Pkl1 asymmetrically blocks microtubule nucleation *in vivo* in fission yeast and that a kinesin-14 Pkl1 Tail peptide can similarly prevent nucleation and generate mitotic arrest in two human breast cancer cell lines. We reveal that, in fission yeast, kinesin-5 Cut7 counters Pkl1 ability to block nucleation by also associating with γ-TuRC and binding similarly to γ-tubulin. This counteraction requires the additional conserved kinesin-5 BimC domain. Balanced regulation by kinesin-14 Pkl1 and kinesin-5 Cut7 generates optimal mitotic fidelity, although both proteins are co-dispensable as determined by genetic analysis of single and double mutants, biochemical approaches and timelapse fluorescence microscopy. Analysis of *pkl1Δ* single and *pkl1Δ cut7Δ* double mutants also reveals separate mitotic roles for both kinesin-14 Pkl1 and kinesin-5 Cut7. Our findings identify kinesin-14 Pkl1 as a Klp-negative regulator of microtubule nucleation at γ-TuRC and demonstrate conservation of this mechanism in human breast cancer cells, resulting in mitotic arrest. We expect these discoveries to be broadly relevant to the microtubule cytoskeleton field with potential as a novel strategy and target for future development in cancer therapeutics.

## Results

### Kinesin-5 is dispensable in the absence of kinesin-14 Pkl1

**Figure 1:**
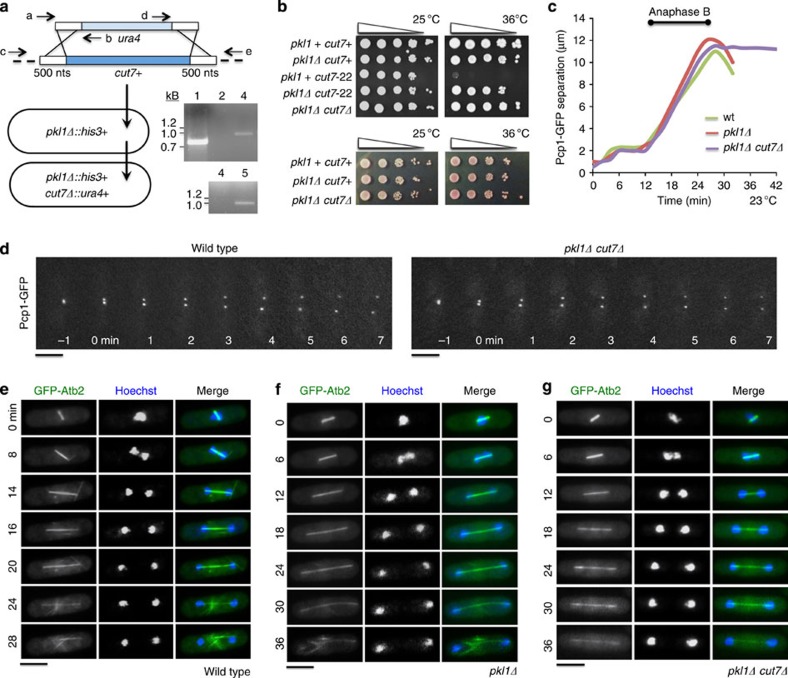
Spindle assembly and cell viability remain high in the *pkl1Δ cut7Δ* strain. (**a**) *cut7* knockout and integration of *ura4+* at this locus in the *pkl1Δ::his3+* single mutant. The PCR-based approach used long (500 nts) flanking tracts of homology and the genomic deletion/integration event was confirmed by colony PCR (lane 3: 5′, lane 5: 3′ oligonucleotide pairs b and c, d and e, respectively). Lane 1 is a positive control using oligonucleotide pair a and b. Lanes 2 and 4 are negative controls using oligonucleotide pair b and c on *pkl1Δ* single-mutant cells. (**b**) Serial dilution growth assays at 25 °C permissive and 36 °C restrictive temperatures (top). Cells were plated on rich YES plates at increasing dilution. Cell viability was analysed by Phloxine B stain (bottom). (**c**) Average spindle pole body separation versus time (Pcp1-GFP) for wild type (green curve; *n*=5 time series), *pkl1Δ* single mutant (red curve; *n*=7 time series) and *pkl1Δ cut7Δ* double mutant cells (purple curve; *n*=9 time series) following hydroxyurea synchronization. (**d**) Timelapse fluorescence microscopy of prophase spindle pole body separation in wild type and *pkl1Δ cut7Δ* cells using spindle pole marker Pcp1-GFP. Timelapse fluorescence microscopy of mitosis from metaphase is shown for wild type in (**e**), *pkl1Δ* in (**f**) and *pkl1Δ cut7Δ* in (**g**). GFP-Atb2 marks microtubules (green) and DNA is stained with Hoechst (blue). Scale bar, 5 μm.

Spindle bipolarity in fission yeast requires kinesin-5 Cut7 (ref. 27). The mechanism underlying its essential nature remains unclear as another Klp, kinesin-6 Klp9, is capable of crosslinking antiparallel microtubules and is required for spindle elongation^28^. In eukaryotes, an opposing relationship between kinesin-5 and kinesin-14 Klps in microtubule regulation is highly conserved. We previously demonstrated that kinesin-14 Pkl1 directly binds and downregulates γ-TuRC function^17^,^23^. We tested the hypothesis that a required role of kinesin-5 Cut7 (*cut7* gene), which localizes at spindle poles, is to oppose kinesin-14 Pkl1 (*pkl1* gene). By homologous recombination (Fig. 1a), we simultaneously deleted the *cut7* gene while marking the locus with *ura4* (*cut7Δ::ura4+*) in a strain deleted for Pkl1 (*pkl1Δ::his3+*). The *pkl1Δ cut7Δ* double mutant strain exhibits robust viability by serial growth assays, similar to wild-type cells (Fig. 1b). Spindle pole body (SPB) separation is not affected in the double mutant *pkl1Δ cut7Δ* versus wild type (Fig. 1c,d) nor is mitotic progression through anaphase affected as compared with wild type or *pkl1Δ* strains (Fig. 1c,e–g). However, spindle breakdown is delayed as indicated by a persistent spindle following anaphase B elongation (Fig. 1c,g). We demonstrate that kinesin-5 Cut7 is dispensable in the absence of Pkl1, indicating that a kinesin-5-independent mechanism for spindle assembly can exist in fission yeast. This also supports a required role for Cut7 that is to counter Pkl1, which is a direct negative regulator of the γ-TuRC MTOC.

### Kinesin-5 Cut7 binds γ-TuRC through Motor and Tail domains

**Figure 2:**
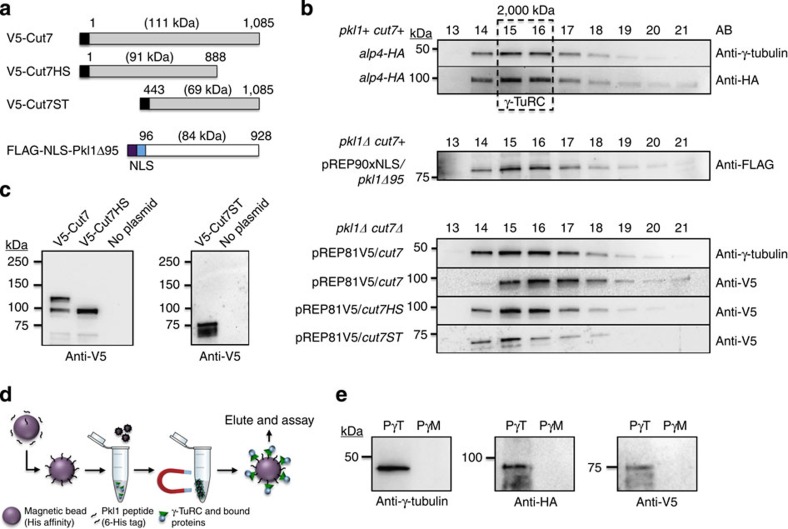
Kinesin-5 Cut7 binds the γ-TuRC MTOC. (**a**) Kinesin-5 and kinesin-14 constructs used in Fast Protein Liquid Chromatography. V5-tagged Cut7 and two truncation constructs were used, in addition to one FLAG-Pkl1 truncated construct that retains full Pkl1 activity. Cut7 constructs are V5-tagged full-length Cut7 (aa 1–1,085), Cut7-Head-Stalk (Cut7HS, aa 1–888) and Cut7-Stalk-Tail (Cut7-ST, aa 443–1,085). (**b**) Western blot profiles of whole-cell extracts fractionated by Separose 6 using FPLC. (**c**) Western blots of Cut7 constructs immunoprecipitated from whole-cell extracts using anti-V5 magnetic beads with empty strain negative controls. (**d**) Cartoon diagram of 6-His tagged Pkl1 Tail peptide co-immunoprecipitation assay using magnetic beads with His affinity and FPLC fraction 15. (**e**) Pkl1 Tail peptide co-immunoprecipitation of γ-TuRC core subunits and V5-Cut7ST using a short Pkl1 Tail peptide (PγT). Mutated peptide PγM has significantly reduced interaction with the fission yeast γ-TuRC. The anti-HA antibody detects the HA-tagged γ-TuRC protein Alp4.

**Figure 3:**
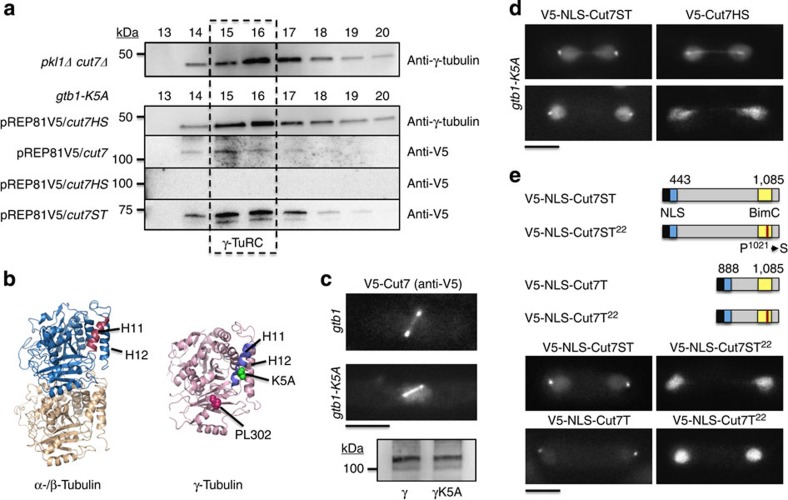
Distinct binding of kinesin-5 Cut7 Motor or BimC domains is required for γ-TuRC association. (**a**) FPLC profiles of V5-tagged Cut7 and two truncation constructs in γ-tubulin helix 11 mutant *gtb1-K5A*. (**b**) Structural model of γ-tubulin-K5A and -PL302 mutants (right) shown with respect to the α-/β-tubulin heterodimer (left). β-tubulin helix 11 is a conserved docking site for Klp Motor domains, and is additionally conserved with fission yeast γ-tubulin helix 11. (**c**) Fluorescence localization and steady-state expression levels from whole-cell extract of full-length V5-Cut7 in wild-type *gtb1* versus the *gtb1-K5A* mutant. (**d**) Fluorescence localization of V5-NLS-Cut7ST (Cut7ST, aa 443–1,085) and V5-Cut7HS in the *gtb1-K5A* strain. (**e**) Fluorescence localization of four *cut7* deletion and BimC site-directed mutagenesis derivatives generated in this study in *pkl1Δ cut7Δ* cells fixed at 36 °C. Deletion constructs used are V5-tagged NLS-Cut7-Stalk-Tail, NLS-Cut7-Stalk-Tail^22^ (Cut7ST^22^, Pro to Ser at aa 1,021), NLS-Cut7-Tail (Cut7T, aa 888–1,085) and NLS-Cut7-Tail^22^ (Cut7T^22^, Pro to Ser at aa 1,021). Scale bars, 5 μm.

Kinesin-14 Pkl1 negatively regulates γ-TuRC through two internal protein domains that include elements of its Motor and Tail regions^17^,^22^. To determine whether Cut7 binds the γ-TuRC MTOC by a similar mechanism, we performed Fast Protein Liquid Chromatography (FPLC)^15^,^17^ using V5-tagged deletion derivatives of Cut7 previously generated^29^, immunocytology using newly generated V5-tagged deletion and site-directed mutagenesis derivatives, and Pkl1 peptide co-immunoprecipitation assays (Fig. 2). Fractionation of whole-cell extracts carrying V5-tagged full-length Cut7 and two Cut7 truncation constructs (Cut7-Head-Stalk or Cut7HS, aa 1–88; Cut7-Stalk-Tail or Cut7-ST, aa 443–1,085) were examined in the *pkl1Δ cut7Δ* double mutant and in the *gtb1-K5A* strain that inhibits Pkl1 regulation of γ-TuRC by blocking its Motor domain binding to γ-tubulin (Figs 2a,b and 3a–c)^22^. A FLAG-tagged truncated Pkl1 construct that retains full function and localizes to γ-TuRC (*pkl1Δ95*)^23^ was used as a positive control along with Alp4 (gamma complex protein GCP2 yeast orthologue) and γ-tubulin proteins that are core subunits of the γ-TuSC and the >2,000 kDa high-molecular weight γ-TuRC MTOC. The γ-TuRC peaks in FPLC fractions 15 and 16 (Fig. 2b). Profiles of the three *cut7* constructs in *pkl1Δ cut7Δ* double mutant cells that are pREP81V5/*cut7*, pREP81V5/*cut7HS* and pREP81V5/*cut7ST* are shown (Fig. 2a,b). Western blot analysis following FPLC fractionation reveals that Cut7, Cut7HS and Cut7ST exhibit similar high-molecular weight profiles as Pkl1Δ95 and all peak in identical fractions to core γ-TuRC proteins γ-tubulin and Alp4. To further confirm Cut7ST binding to γ-TuRC, we performed co-immunoprecipitations from high-molecular weight FPLC fraction 15 in strain *alp4-HA* pREP81V5*/cut7ST* (Fig. 2d,e). Magnetic beads with histidine affinity were conjugated to small His-tagged Pkl1 Tail peptides (PγT or PγM)^17^ that bind γ-TuRC (PγT, targeting) or cannot interact with fission yeast γ-TuRC (PγM, mutated). Using this approach, we detect γ-TuRC core subunit proteins γ-tubulin and Alp4-HA in addition to Cut7ST (V5 tag) by western blot analysis after elution off of beads (PγT). These proteins were not recovered by the mutated peptide, as expected. We were unable to detect α-/β-tubulin in high-molecular weight fractions following FPLC, which further suggests that the Klps detected were directly bound to γ-TuRC.

We previously demonstrated that mutation of a conserved lysine residue to alanine in γ-tubulin helix 11 (*gtb1-K5A*) abolishes Pkl1 Motor domain binding to γ-TuRC and blocks its full function *in vivo*^22^. To determine whether Cut7 similarly binds to γ-tubulin through helix 11, we examined its FPLC profile in the *gtb1-K5A* strain (Fig. 3a). The mutant γ-tubulin-K5A fractionates similarly to wild-type γ-tubulin by FPLC. The V5-Cut7 signal in high-molecular weight γ-TuRC fractions is significantly reduced in the *gtb1-K5A* strain, whereas steady-state expression levels of V5-Cut7 in *gtb1* wild type and *gtb1-K5A* mutant backgrounds by western blot analysis of whole-cell extracts is similar (Fig. 3c). Fluorescence microscopy of V5-Cut7 in the *gtb1-K5A* mutant reveals that, while pole localization is reduced, Cut7 retains localization to spindle microtubules, which is enhanced. Truncated Cut7HS that contains the Cut7 Motor and Stalk domains and lacks the Tail is completely absent from high-molecular weight γ-TuRC fractions in the *gtb1-K5A* background (Fig. 3a). Similarly to V5-Cut7, it retains the ability to bind to spindle microtubules as observed by immunocytology (Fig. 3d). Interestingly, the truncated Cut7ST protein in this strain that does not retain the Motor domain is unaltered in its association with γ-TuRC (Fig. 3a). The V5-Cut7ST with a fused nuclear localization single (NLS) to allow nuclear localization^29^ is sufficient to target spindle poles in mitosis (Fig. 3d). This indicates that, similar to Pkl1, Cut7 contains distinct Motor and Tail domain elements that offer independent binding sites to γ-TuRC.

### The kinesin-5 Cut7 BimC Tail element directs pole targeting

The eukaryotic BimC domain is highly conserved across kinesin-5 members^30^,^31^. The domain was first identified in the *Aspergillus nidulans* kinesin-5 BimC protein^32^, but its precise role in mitosis has remained unknown for two decades. To examine the consequences of a mutation to the BimC domain of Cut7 on spindle pole localization, we used the loss of function temperature sensitive allele *cut7-22* that contains a single-point mutation within this region (1,021 Pro to Ser)^22^. To determine whether the BimC box is the primary spindle pole targeting site in the Cut7 Tail domain, we utilized V5-tagged Cut7 deletion and/or site-directed mutagenesis constructs with a fused NLS (Fig. 3e)^23^. These constructs encode the fusion proteins V5-NLS-Cut7ST, V5-NLS-Cut7ST^22^ (point mutation at 1,021 Pro to Ser resulting in lost bipolarity)^22^, V5-NLS-Cut7T (Cut7-Tail, aa 888–1,085) and V5-NLS-Cut7T^22^ and were analysed by immunocytochemistry of *pkl1Δ cut7Δ* cells that were fixed after shifting to 36 °C. V5-NLS-Cut7ST and V5-NLS-Cut7T lack the Motor domain and retain spindle pole localization. In contrast, mutated V5-NLS-Cut7ST^22^ and V5-NLS-Cut7T^22^ constructs are unable to localize to poles but are expressed and retained within the nucleus, suggesting that the BimC sequence constitutes a key domain in the Cut7/γ-TuRC interaction. Together, our data support the model in which Cut7 interacts physically with the γ-TuRC MTOC in a manner poised to allow opposition to Pkl1 activity.

### Pkl1 regulates spindle morphology from γ-TuRC

**Figure 4:**
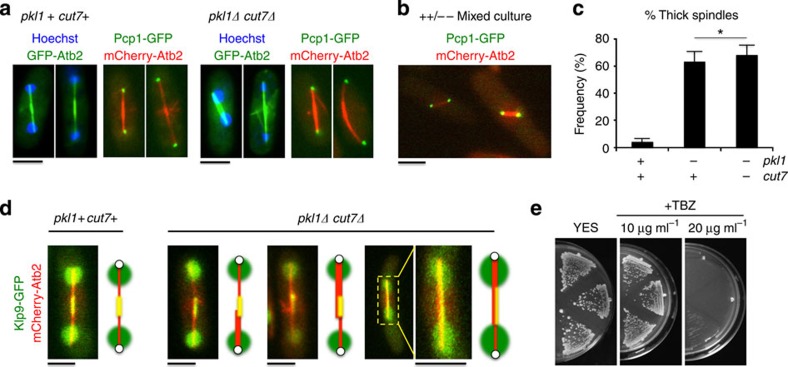
Spindle width is increased in *pkl1Δ* and *pkl1Δ cut7Δ* cells. (**a**) Live cell fluorescence imaging reveals differences in spindle thickness between wild type (*pkl1+ cut7+*) and *pkl1Δ cut7Δ* double mutant cells. Two stages of mitosis are shown with different markers. On the left, GFP-Atb2 marks microtubules (α-tubulin, green) and DNA is stained with Hoechst (blue). On the right, microtubules are marked by mCherry-Atb2 (red) and spindle pole bodies are marked by Pcp1-GFP (green). (**b**) Differences in spindle thickness in a wild type/*pkl1Δ cut7Δ* mixed culture by live cell fluorescence microscopy. (**c**) Frequency of thick spindles in wild type, *pkl1Δ* and *pkl1Δ cut7Δ* cells (mean±s.e.m., *n*=90 cells for each, *P<0.05 by Student’s *t*-test). (**d**) Live cell fluorescence microscopy of wild type and *pkl1Δ cut7Δ* cells with mCherry-Atb2 and Klp9-GFP suggests that the increased spindle thickness we observe is due to parallel microtubules that emanate from a single pole (highlighted by cartoon schematics). Klp9-GFP marks antiparallel microtubules at the spindle midzone. In the schematic, yellow marks Klp9-GFP/antiparallel microtubule overlap, red marks parallel microtubules that extend from either pole (white circle), and green is Klp9-GFP signal on chromatin. Images were oriented similarly for convenience. Similar results to *pkl1Δ cut7Δ* cells were observed for the *pkl1Δ* single mutant. (**e**) Plating of wild type, *pkl1Δ* and *pkl1Δ cut7Δ* (top to bottom) cells on medium containing three concentrations of the microtubule-depolymerizing drug TBZ (0 μg ml^−1^ left, 10 μg ml^−1^ middle, 20 μg ml^−1^ right). Scale bar, 5 μm.

Mitotic phenotypes in strains carrying single *pkl1Δ* or *pkl1Δ cut7Δ* double mutants versus wild type were evaluated by live cell and timelapse imaging of microtubules (α-tubulin as mCherry-Atb2 or GFP-Atb2), spindle poles (γ-TuRC pericentrin protein 1, Pcp1-GFP), antiparallel microtubules and chromatin (anaphase B and chromatin binding kinesin-6 member Klp9-GFP) as well as Hoechst staining of DNA. An increase in spindle thickness is observed in the *pkl1Δ cut7Δ* double mutant strain versus wild type as seen in single-strain imaging (Fig. 4a) or by live cell imaging of a mixed culture with wild-type cells (Fig. 4b). This increased thickness is also present in the *pkl1Δ* strain (Fig. 4c). In Fig. 4b, the ratio of double mutant to wild-type cells at 2:1 resulted in an accordant increase in the ratio of thick:thin spindles. Thick spindles (>0.5 μm in a spindle of length 4 to 6 μm) are observed in 4±2% of wild-type cells, 63±8% *pkl1Δ* single mutant and 68±8% of *pkl1Δ cut7Δ* double mutant cells (mean±s.e.m., *n*=90 cells for each strain). Although the *pkl1Δ cut7Δ* double mutant background generates a slight increase in the thick spindle phenotype (*P*<0.05 by Student’s *t*-test), the change versus *pkl1Δ* single mutant alone is small. This indicates that it is primarily the loss of Pkl1 that induces this phenotype, which is exacerbated by additional loss of Cut7.

The nature of the morphological change to spindle thickness could be the result of an increase in the number of antiparallel microtubules from both poles, parallel microtubules that emanate from a single pole, or due to unattached microtubules or disorganized arrays^20^ at a single pole. To distinguish among these possibilities, we used multiple approaches. kinesin-6 Klp9-GFP crosslinks antiparallel microtubule arrays^28^ and can be used to preferentially mark the extent of microtubule overlap (generally midzone), and is used with α-tubulin (mCherry-Atb2) to visualize spindle microtubules and length of the mitotic spindle. In wild-type cells, the Klp9-GFP signal spans the entire spindle midzone width (Fig. 4d, left images), whereas in the double mutant (Fig. 4d, right images) we observe microtubule staining adjacent to the zone of antiparallel overlap that appears to emanate primarily from a single pole. We do not detect increased resistance to the microtubule-depolymerizing drug Thiabendazole (TBZ) in *pkl1Δ* or *pkl1Δ cut7Δ* strains versus wild type (Fig. 4e) consistent with no or limited changes to microtubule number. We favour the interpretation that increased spindle width is likely due to asymmetric spindle pole effects (lost organization) in the absence of Pkl1 as seen^20^ and to an increased number of spindle microtubules.

**Figure 5:**
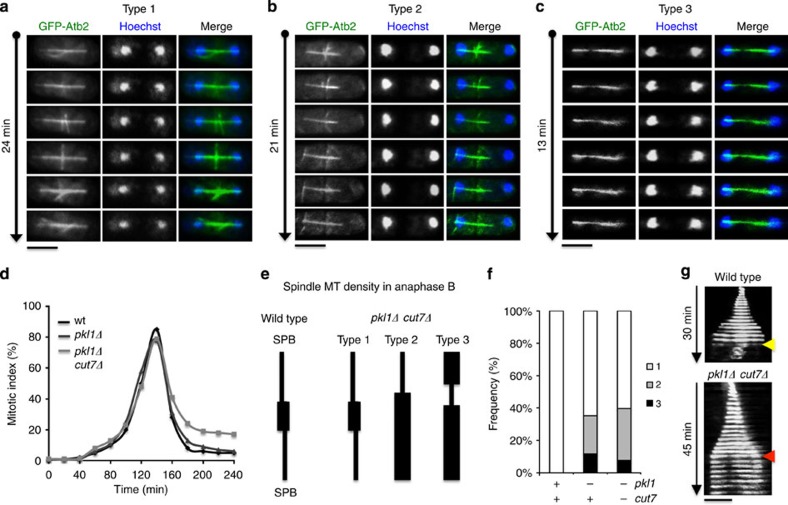
Spindle breakdown is delayed in the *pkl1Δ cut7Δ* double mutant. (**a**–**c**) Timelapse fluorescence microscopy of persistent anaphase B spindles in double mutant cells. Three types of spindle microtubule density were observed, shown in (**e**). (**d**) Mitotic index versus time for wild type (black curve), *pkl1Δ* (dark grey curve) and *pkl1Δ cut7Δ* cells (light grey curve) following hydroxyurea arrest and release. Spindle length was measured using microtubule stain by ICC. (**e**) Schematic of three types of anaphase spindle microtubule density observed in *pkl1Δ cut7Δ* double mutant cells. (**f**) Stacked histogram representation of spindle microtubule density phenotypes across strains. *n*=300 cells averaged over three time points per strain. (**g**) Kymographs of a wild-type spindle (top; yellow arrow indicates spindle breakdown) and a persistent spindle (red arrow) shown from the time series for Type 3. Scale bar, 5 μm.

Morphological changes in spindle thickness do not affect mitotic progression in the *pkl1Δ cut7Δ* double mutant cells through anaphase as seen by timelapse imaging and kymographic analysis versus wild type (Fig. 5). However, following anaphase B, timely spindle breakdown is delayed in a significant percentage of *pkl1Δ cut7Δ* cells. In 78% of double mutant cells (*n*=15 timelapse series) spindles remain intact significantly longer than wild type (*n*=7 timelapse series) for an additional 24±7 min, and can persist beyond formation of equatorial MTOC arrays (Fig. 5a–c). Three patterns of spindle microtubule density in *pkl1Δ cut7Δ* anaphase cells were observed (Fig. 5a–c,e) and are referred to as Type 1, Type 2 and Type 3. In Type 1, central microtubule antiparallel overlap is identical to wild type. In Type 2, microtubule density is highly biased to one pole, and, in Type 3, central microtubules are diminished compared with thicker pole-biased microtubules. The relative frequencies of these patterns averaged over three time points after hydroxyurea synchronization (120, 140 and 160 min) are indicated in a stacked histogram (Fig. 5f). Our findings indicate that changes occur to spindle width and organization in both the *pkl1Δ cut7Δ* double mutant and *pkl1Δ* single mutant strains. Thickness along the spindle length is distributed in three patterns, two that are distinct from wild type. Spindle morphology phenotypes in the *pkl1Δ cut7Δ* double mutant are only modestly altered versus the single *pkl1Δ* mutant strain. These findings indicate that kinesin-14 Pkl1 is the primary kinesin regulating microtubule organization at γ-TuRC, a loss that results in broader spindles with asymmetric microtubule density along the spindle length.

### Daughter pole disorganization persists in the double mutant

**Figure 6:**
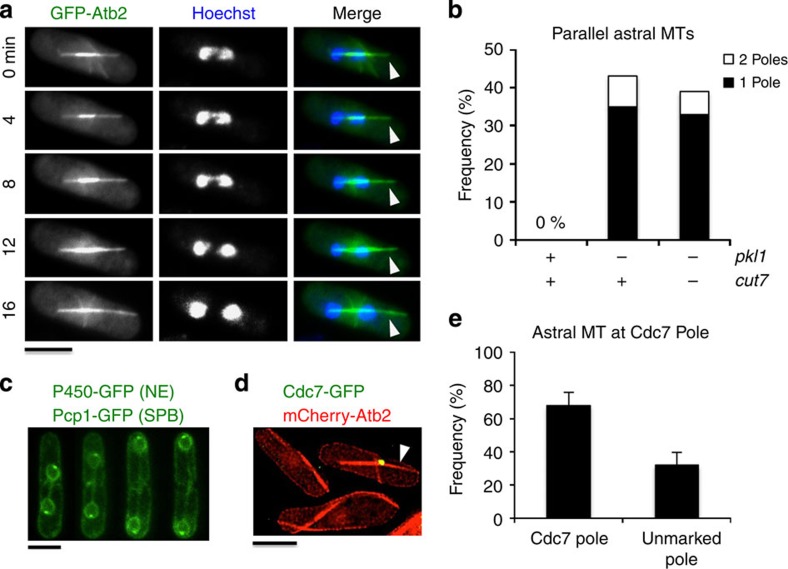
Loss of Pkl1 affects daughter pole organization. (**a**) Live cell timelapse series showing asymmetric astral microtubule array parallel to the mitotic spindle (white arrows) in the *pkl1Δ cut7Δ* double mutant. GFP-Atb2 marks microtubules (green) and DNA is stained with Hoechst (blue). (**b**) Frequency of parallel symmetric versus parallel asymmetric astral microtubule arrays in three strains. Only cells that had astral microtubule arrays were included in statistical analysis (*n*=45 cells per strain). (**c**) Spindle pole body (Pcp1-GFP) and nuclear envelope (NE; P450-GFP) markers in a mitotic *pkl1Δ cut7Δ* cell do not exhibit abnormal NE protrusions beyond either pole (*n*=0/57 mitotic cells). (**d**) Cdc7-GFP is an asymmetric pole marker that localizes primarily to the daughter pole in mitosis. Asymmetric astral microtubule arrays parallel to the mitotic spindle in *pkl1Δ cut7Δ* cells extend primarily from the pole marked by Cdc7-GFP (white arrow; *n*=27 cells). Scale bar, 5 μm. (**e**) Comparison of parallel astral microtubules that extend from a pole marked by Cdc7-GFP versus the unmarked pole in *pkl1Δ cut7Δ* double mutant (*n*=45 cells).

An asymmetric effect on SPB organization with loss of the typical plaque-like appearance at one pole has been observed by TEM analysis of the *pkl1Δ* strain^20^. Similarly in the *pkl1Δ cut7Δ* double mutant we observe an asymmetric effect on spindle poles, including altered astral microtubule arrays as previously shown^33^. Here we additionally identify the daughter pole as being primarily affected (Fig. 6). Asymmetry in astral microtubule lengths from opposing poles is observed and orientation is parallel to the spindle axis beginning in early mitosis. Asymmetric astral arrays are observed in 35±6% of *pkl1Δ* cells and 33±6% of *pkl1Δ cut7Δ* cells (Fig. 6b; mean±s.e.m., *n*=200 cells per strain). A small percentage of cells in both strains (8±3% and 6±2%, respectively) exhibits parallel arrays that are symmetric, and the remainder of cells examined have normal appearing astral arrays. Cells without astral microtubules were also observed, but excluded from this analysis. We do not observe protrusions in the nuclear envelope as observed by co-imaging with nuclear envelope and SPB markers shown in Fig. 6c (*n*=0/57 cells), suggesting that these arrays are cytoplasmic. The septation-initiation network (SIN) protein Cdc7 loads primarily to the daughter pole in mitosis^34^,^35^. We found that the longer abnormal astral microtubules extend from poles that harbour Cdc7-GFP (68±8% of cells with a signal, *n*=29 cells) and that this is also the pole with increased thickness (Fig. 6a,d). These data extend the asymmetric SPB disorganization phenotype observed in *pkl1Δ* cells^20^, identifying a role for Pkl1 in maintaining daughter pole organization from γ-TuRC that indirectly alters astral microtubule arrays in the cytoplasm. Further, the data are consistent with daughter pole disorganization contributing to the thicker spindle phenotypes we observe in both *pkl1Δ* and *pkl1Δ cut7Δ* mutant cells.

### *pkl1Δ* and *pkl1Δ cut7Δ* share chromosome segregation defects

**Figure 7:**
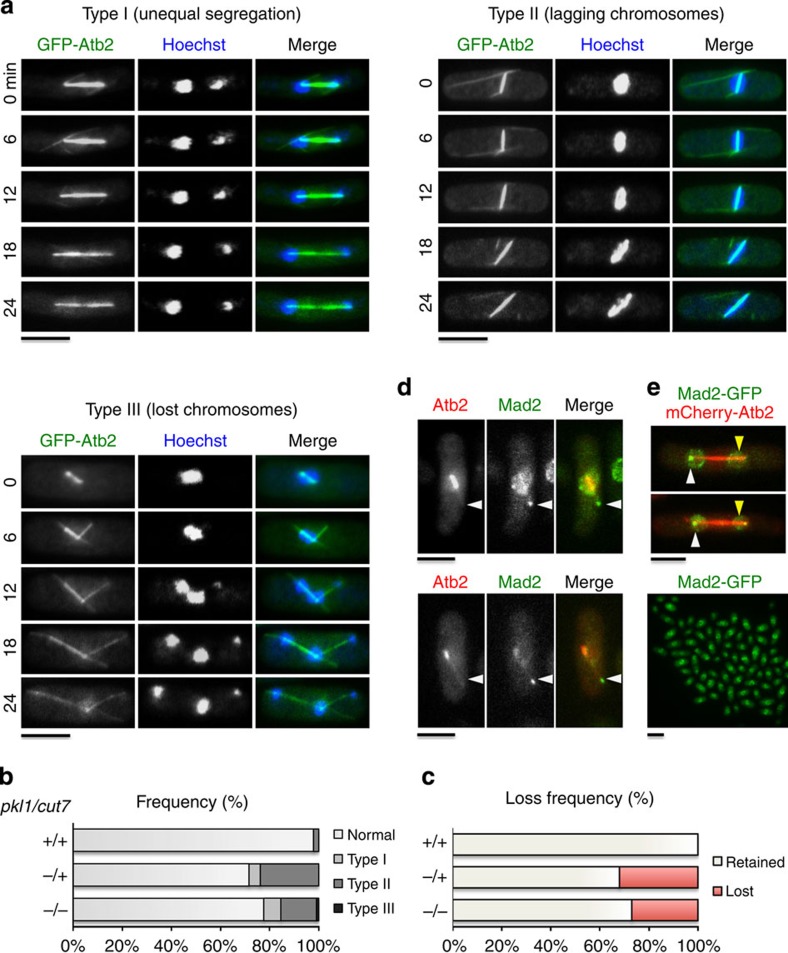
Double mutant cells exhibit defective chromosome segregation. (**a**) Three live cell timelapse series showing chromosome missegregation in *pkl1Δ cut7Δ* double mutant cells. (**b**) Frequency of missegregation phenotypes across strains (*n*=500 cells/strain). (**c**) Mini chromosome loss frequency in wild type (*n*=0/1,011, 0%), *pkl1Δ* single mutant (*n*=316/986, 32%) and *pkl1Δ cut7Δ* double mutant cells (*n*=549/2,035, 27%). (**d**) *pkl1Δ cut7Δ* cells expressing Mad2-GFP (green) and mCherry-Atb2 (red). (**e**) Increased spindle microtubule density at one pole (yellow arrow) in *pkl1Δ cut7Δ* double mutant cells is associated with little to no Mad2-GFP polar signal in anaphase B (top images). The white arrow indicates the mother pole. Mad2-GFP is stably expressed in *pkl1Δ cut7Δ* cells (bottom image). In this bottom image, scale bar, 10 μm. Scale bar, 5 μm.

We monitored chromosome segregation in the double mutant by live cell timelapse fluorescence microscopy and immunocytology of microtubules and DNA, as well as mini chromosome loss (Fig. 7). Three types of chromosome segregation defects are present in the double mutant that are unequal segregation, lagging chromosomes and lost chromosomes (Fig. 7a). Compared with wild-type cells, the *pkl1Δ cut7Δ* double mutant strain has increased lagging chromosomes and unequally segregated chromosomes, as well as a minor increase in lost chromosomes^36^. However, we along with others^20^ observe that the efficiency of chromosome segregation is already markedly reduced in the absence of kinesin-14 Pkl1. Compared with the *pkl1Δ cut7Δ* double mutant strain, no lost chromosomes are observed in *pkl1Δ* and unequal segregation is reduced, though lagging chromosomes are prominent. Figure 7b is a histogram representation of the relative frequencies of these phenotypes in wild type, *pkl1Δ* single mutant and *pkl1Δ cut7Δ* double mutant strains. Shown are frequencies (*n*=500 cells) for unequal segregation (Type 1; wild type: 0%, *pkl1Δ*: 7±3% and *pkl1Δ cut7Δ*: 9±3%, mean±s.e.m.), lagging chromosomes (Type 2; 3±2, 23±5 and 30±5%) and chromosome loss from the spindle (Type 3; 0, 0 and 1%). We further quantified chromosome missegregation by monitoring the loss rate of a mini chromosome (*cen1-7L*) *sup3E* in the three strains (Fig. 7c). Mini chromosome loss in the *pkl1Δ cut7Δ* double mutant is greater than that in wild type (27% increase) but reduced by 5% with respect to *pkl1Δ* (32%). Our findings on chromosome segregation with *pkl1Δ* are consistent with previous studies^20^ and reveal both rescue (lagging chromosomes) and exacerbation (unequal segregation) of the *pkl1Δ* phenotype in the double mutant along with the additional lost chromosome phenotype.

In wild-type cells, Mad2 monitors proper bipolar attachment of spindle microtubules to the kinetochore (KT), then transitions from KTs to both SPBs coincident with anaphase A onset. In anaphase B, Mad2 becomes asymmetric and makes a subsequent transition from the daughter pole to the equatorial MTOC, but remains asymmetrically localized at the mother pole^26^. We observe Mad2-GFP associated with the lost chromosome that is attached by an intranuclear microtubule (Fig. 7d), similar to what was observed previously with the lost chromosome phenotype^36^. This lost chromosome event occurs in 1% of *pkl1Δ cut7Δ* cells (*n*=3/300 cells) and is not observed in *pkl1Δ* cells. Mad2-GFP was also useful in confirming that the increased spindle microtubule thickness we observe in Type 2 morphologies (Fig. 5b,e) is associated with the daughter pole. By imaging cells with Mad2-GFP and mCherry-Atb2, the increased spindle microtubule thickness extends primarily from the daughter spindle pole that is unmarked or dimly marked by Mad2-GFP in early anaphase B (Fig. 7e), consistent with our Cdc7-GFP data (Fig. 6). Our findings indicate that the chromosome segregation defects in the double mutant strain generally resemble those of *pkl1Δ* except for the additional presence of the rare lost chromosome phenotype.

### An asymmetric block on γ-TuRC nucleation competency by Pkl1

**Figure 8:**
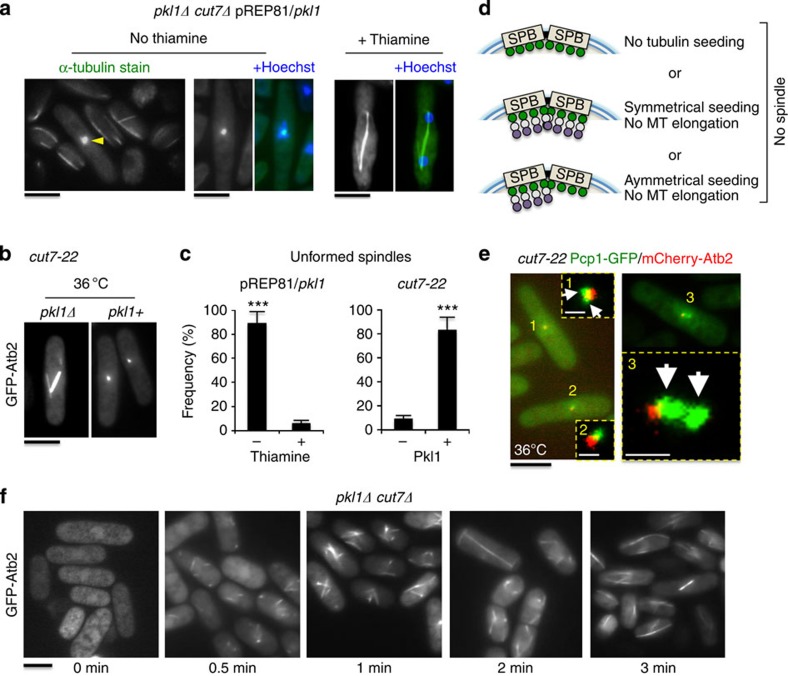
Pkl1 opposes γ-TuRC microtubule nucleation asymmetrically from spindle poles. (**a**) Pkl1 inhibits spindle formation in *pkl1Δ cut7Δ* double mutant cells. Cells were inoculated from thiamine plates into selective media containing 0 μg ml^−1^ or 5 μg ml^−1^ thiamine for nmt promoter repression or induction, respectively, and were fixed with methanol after 17 h. Samples were stained with anti-tubulin TAT1 antibody and DNA was stained with Hoechst (*n*=200 cells for both conditions). (**b**) Pkl1 inhibits mitosis in *cut7-22* temperature sensitive cells at 36 °C. *pkl1+ cut7-22* and *pkl1Δ cut7-22* cells expressing GFP-Atb2 were synchronized in hydroxyurea for 4 h at 27 °C permissive temperature, released at 36 °C in minimal supplemented medium and imaged 4 h after release (*n*=200 cells per strain). (**c**) Frequency of unformed spindles in (**a**) and (**b**). ***P<0.001 by Student’s *t*-test (mean±s.e.m.). (**d**) Cartoon diagram of three possibilities for MTOC tubulin seeding at poles that would not permit spindle assembly. γ-TuRC is in green and the α-/β-tubulin heterodimer is shown in white/purple. Nuclear envelope is shown in blue. (**e**) To distinguish between the possibilities in (**c**), *pkl1+ cut7-22* cells expressing mCherry-Atb2 (red) and pole marker Pcp1-GFP (green) were analysed by the experiment used in (**b**). Zoomed-in images are high contrast maximum intensity Z-stack projections generated in ImageJ. White arrows highlight distinct poles, and white scale bars are 1 μm. (**f**) Microtubule depolymerization by cold shock, nucleation and repolymerization at 32 °C in double mutant cells. *pkl1Δ cut7Δ* cells with integrated GFP-Atb2 were fixed at the designated time points to preserve GFP signal. All black scale bars in this figure are 5 μm.

We previously demonstrated that kinesin-14 Pkl1 physically interacts with γ-TuRC in prophase to downregulate its function and oppose bipolar spindle assembly^17^,^22^. We established an *in vivo* assay system to examine the impact of elevated levels of Pkl1 in double mutant cells where kinesin-5 Cut7 is absent (Fig. 8a). Multi copy *pkl1* was expressed using the low strength thiamine repressible nmt promoter (pREP81/*pkl1*). The *pkl1Δ cut7Δ* cells containing pREP81/*pkl1* were grown with or without 5 μg ml^−1^ thiamine and were fixed after 17 h. Cells were stained for α-tubulin and DNA using TAT1 antibody and Hoechst, respectively (Fig. 8a). After thiamine release we observed that 89±9% of cells were arrested with unformed spindles. This frequency was only 6±2% when cells were grown in the presence of thiamine (Fig. 8c, left plot; mean±s.e.m., *n*=200 cells for both conditions). In cells with unformed spindles, tubulin signal was concentrated near the cell centre. We were unable to obtain *pkl1Δ cut7Δ* cells transformed with *pkl1* expressed under a higher strength promoter (pREP90x/*pkl1*), even under promoter repressing conditions in the presence of thiamine. We additionally performed live cell fluorescence microscopy with GFP-Atb2 (α-tubulin) in the *cut7-22* temperature sensitive strain with or without the native *pkl1* gene (Fig. 8b). Cells were synchronized by hydroxyurea for 4 h, released at the 36 °C restrictive temperature and imaged 4 h after release. We observed similar unformed spindles in 82±9% of cells containing Pkl1. The *pkl1Δ cut7-22* cells grown similarly exhibit robust spindle assembly (Fig. 8c, right plot; *n*=200 cells for both conditions), and these data are consistent with the serial growth assays performed with these two strains at 36 °C (Fig. 1b).

We envision three possible models in which spindle assembly would fail via a spindle pole based mechanism that are based on tubulin seeding at γ-TuRC (Fig. 8d). This includes no addition of tubulin to γ-TuRC, symmetrical addition but without microtubule elongation, or asymmetric addition of tubulin at a single spindle pole. Our data indicate the presence of short microtubules, ruling out model 1. To distinguish between models 2 and 3, we generated the strain [*pkl1+ cut7-22 pcp1-GFP mCherry-atb2*] to simultaneously visualize poles and microtubules (compare Fig. 8b with Fig. 8e). Our data support model 3. We observe that in 100% of arrested cells where two distinct poles could be identified, the tubulin signal was associated with a single pole (*n*=25 cells). *In vivo* nucleation shown by cold depolymerization and reformation of microtubules in Fig. 8f demonstrates that, in *pkl1Δ cut7Δ* cells, γ-TuRC can nucleate both interphase and spindle microtubules, consistent with our other data. Together, the *in vivo* data here are consistent with our model in which a required role for Cut7 in spindle assembly is to bind γ-TuRC to oppose Pkl1 activity. Therefore, of these Klps, kinesin-14 Pkl1 in fission yeast is the primary negative regulator of γ-TuRC microtubule nucleation.

### Kinesin-14 PγTR peptide arrests human breast cancer cells

**Figure 9:**
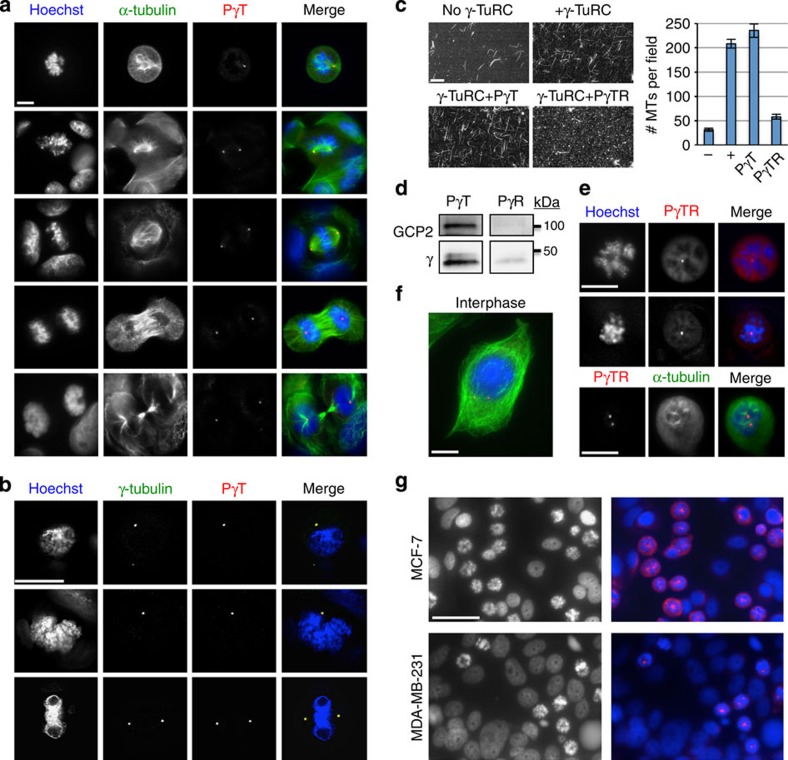
Yeast γ-TuRC peptide PγTR causes mitotic arrest in human breast cancer cells. Conserved action of the minimal Pkl1 Tail domain. (**a**) Localization of γ-TuRC targeting peptide PγT to centrosomes in fixed human MCF-7 breast cancer cells. DNA is shown in blue (Hoechst), microtubules in green (α-tubulin) and PγT peptide in red. 6-His tagged PγTR was administered before addition of primary antibodies. (**b**) Co-localization of γ-tubulin and PγTR to centrosomes in mitotic MCF-7 cells. Scale bars in (**a**) and (**b**) are 10 μm. (**c**) *In vitro* γ-TuRC microtubule nucleation assays. No γ-TuRC negative controls provide background for spontaneous microtubule formation from tubulin (1.5 μg μl^−1^). All other samples use whole-cell extract from human breast cancer cells. γ-TuRC targeting and regulatory peptide PγTR blocks γ-TuRC nucleation efficacy. Scale bar, 20 μm (mean±s.d. for number of microtubules per field, *n*=3 experiments). (**d**) Magnetic bead co-immunoprecipitation of γ-TuRC core proteins GCP2 and/or γ-tubulin using 6-His-tagged PγT (targeting) and PγR (regulatory) peptides (see Fig. 2d for method). (**e**) High-magnification images of MCF-7 cells arrested by PγTR (live cell transfection). DNA is in blue (Hoechst) and PγTR is in red (top). Microtubules are shown in the bottom panel in green. Scale bar, 10 μm. (**f**) Non-arrested MCF-7 cell containing low levels of PγTR. Scale bar, 10 μm. (**g**) Mitotic arrest in MCF-7 (low-aggressiveness; top images) and MDA-MB-231 cells (high aggressiveness; bottom images) 24 h after transfection with 1 μg (108 μM) of γ-TuRC targeting and regulatory peptide, PγTR. At this time point, 43.3% of MCF-7 cells (681/1,572 cells counted from *n*=12 fields at × 200) and 27.7% of MDA-MB-231 cells (497/1,732 cells counted from *n*=12 fields at × 200) were transfected with peptide based on fluorescence staining using the 6-His tag. Of this, 39% of MCF-7 cells (613/1,572) and 22.6% of MDA-MB-231 cells were arrested in mitosis. Left images are Hoechst, and right images are merged Hoechst+PγTR for both cell lines. Scale bar, 50 μm.

All human γ-TuSC proteins are functional in fission yeast^14^,^15^. We previously developed biochemical tools in the form of kinesin-14 Tail peptides (Fig. 2e)^17^ that regulate γ-TuRC *in vitro* and here tested the conserved capability of these peptides to block microtubule nucleation in human MCF-7 breast cancer cells (Fig. 9). By immunocytology in fixed MCF-7 cells we demonstrate that 6-His tagged PγT targets the centrosome (Fig. 9a) and co-localizes with γ-tubulin (Fig. 9b). By microtubule nucleation assays with or without γ-TuRC, we also investigated the effect of kinesin-14 peptides on human γ-TuRC nucleation competency *in vitro* (Fig. 9c). The control targeting peptide PγT is insufficient to block γ-TuRC microtubule nucleation alone. However, PγTR that contains an additional γ-tubulin binding and regulatory sequence^17^ is a potent inhibitor of nucleation. Peptides PγT and PγR (separated TR elements) isolate human γ-TuRC components *in vitro* similar to their action in fission yeast (Figs 2e and 9d). That is, PγT co-immunoprecipitates human GCP2, the yeast Alp4 counterpart, in addition to γ-tubulin, while PγR binds and removes γ-tubulin from the complex^17^.

By live cell transfection of human MCF-7 breast cancer cells that exhibit low aggressiveness or MDA-MB-231 cells that are highly aggressive, we show that PγTR is a potent mitotic spindle protein (MSP) class regulator of mitotic arrest (Fig. 9e–g). Cell lines were transfected with 1 μg of 6-His tagged PγTR in 2 ml (108 μM) using the Chariot system (Active Motif) and fixed after 24 h. At this time point, 43.3% of MCF-7 cells (681/1,572 cells counted from *n*=12 fields at × 200) and 27.7% of MDA-MB-231 cells (497/1,732 cells counted from *n*=12 fields at × 200) were transfected with peptide based on fluorescence staining using the 6-His tag. Of this, 39% of MCF-7 cells (613/1,572) and 22.6% of MDA-MB-231 cells were arrested in mitosis. Cells lacking peptide signal did not arrest. Breast cancer lines transfected with a control 6-His hexamer also did not arrest, indicating that this tag does not contribute to the anti-mitotic effect of PγTR. In a small percentage of the populations (4.3% MCF-7; 5.1% MDA-MB-231) with very low but detectable levels of peptide at the centrosome, cells appeared normal (MCF-7 shown in Fig. 9f). In arrested cells, residual clumped microtubules are present but do not appear to extend from centrosomes, suggesting that PγTR has an inhibitory effect on γ-TuRC microtubule nucleation *in vivo* in human cells. These findings suggest that kinesin-14 action at γ-TuRC is conserved from the yeast SPB to the human centrosome. We expect that PγTR will be a powerful mechanistic tool to elucidate γ-TuRC function for microtubule nucleation in multiple model organisms and a potential therapeutic tool for preventing cell proliferation in disease.

## Discussion

Microtubule organizing centres play major roles in specialized eukaryotic processes of broad interest such as spindle assembly, neuronal function and immunological synapse formation that involves cell polarization. Understanding the detailed mechanisms for microtubule nucleation requires combined knowledge of the underlying structure along with regulatory insights. In this work we demonstrate that the ability of fission yeast kinesin-14 Pkl1 to bind and alter γ-TuRC structure and function^17^ results in blocked microtubule nucleation *in vivo* generating failed spindle bipolarity and mitotic arrest. Conservation of this mechanism is revealed through use of a kinesin-14 Pkl1 peptide PγTR in human breast cancer cells that localizes to centrosomes and is sufficient to arrest nucleation in the two breast cancer cell lines investigated by preventing bipolar spindle formation. In fission yeast, kinesin-5 Cut7 but not kinesin-14 Pkl1, is an essential mitotic protein^18^,^27^. To better understand kinesin-14 Pkl1 function at γ-TuRC, and kinesin-5 counteraction of this Klp, we applied genetic analysis, biochemistry and timelapse imaging. Here we show that kinesin-5 Cut7 is dispensable in the absence of kinesin-14 Pkl1 and that counteraction of Pkl1 by Cut7 requires Cut7 binding to γ-TuRC through its Motor and BimC domains. These Klps are the first identified to directly bind and regulate γ-TuRC, actions that are sufficient to impact microtubule nucleation capability. These findings are expected to have significant impact in the cytoskeleton field, particularly in understanding MTOC function as well as in potential therapeutic anti-cancer applications that utilize mitotic spindle protein agonists.

Distinct mitotic phenotypes are present with loss of either Pkl1 or Cut7. The loss of *pkl1* in the presence of *cut7*, although viable, results in an asymmetric effect on daughter spindle pole organization that influences spindle width and impairs chromosome segregation. Compared with the double mutant *pkl1Δ cut7Δ*, no amelioration or exacerbation of the *pkl1Δ* phenotype is observed, indicating that these phenotypes are likely due to loss of Pkl1. The additional loss of Cut7, however, does result in delayed spindle breakdown for mitotic exit. Cut7 localizes to the spindle midzone in anaphase^37^, and, although not required for anaphase B spindle elongation^29^, our data indicate it may contribute to normal progression through this stage. A primary role for kinesin-5 Cut7 is therefore to counteract kinesin-14 Pkl1 at γ-TuRC. Only in the presence of Pkl1 does removal of Cut7 or inactivation of the Cut7 BimC domain (*cut7-22*) allow an asymmetric block on γ-TuRC microtubule nucleation to be imposed that results in failed spindle bipolarity (Fig. 8).

Extensive studies demonstrate both the importance of kinesin-5 motors in spindle assembly along with kinesin-5-independent mechanisms. In the latter, force generation by other microtubule motors such as nuclear envelope-associated dynein and kinesin-12 operate and include microtubule pushing forces on the opposing pole and kinetochore-mediated microtubule interactions in prophase^38^. Our ability to remove kinesin-5 Cut7 in the absence of kinesin-14 Pkl1 reveals that in fission yeast kinesin-5-independent mechanisms exist to establish spindle bipolarity.

Spindle phenotypes in the double mutant and single *pkl1Δ* strains do not include changes to timing for prophase SPB separation or mitotic progression through anaphase B versus wild type. The increase in spindle thickness on loss of Pkl1 that we observe is reminiscent of phenotypes induced by loss of kinesin-14 Kar3 in budding yeast^19^. The thick spindle morphology did not result in increased resistance to the microtubule-depolymerizing drug TBZ at 10 or 20 μg ml^−1^ concentrations in single or double mutant backgrounds versus wild type, consistent with no change in spindle microtubule number. We favour the model that altered microtubule organization of parallel microtubules emanating from the daughter pole results in increased spindle width at this pole as opposed to an increase in spindle microtubule number. This is consistent with studies by ref. 20 in which TEM analysis of *pkl1Δ* cells revealed a decrease in pole organization characterized by loss of the typical plaque-like structure with apparent normal microtubule number. The replication of the SPB and centrosome is semi-conservative with the mother pole used as a template. To identify whether the mother or daughter pole is affected by loss of *pkl1*, we applied live cell fluorescence microscopy with asymmetric pole markers Cdc7-GFP and Mad2-GFP along with mCherry-Atb2 to mark microtubules. Our studies reveal that the daughter pole is affected in both *pkl1Δ* single mutant and *pkl1Δ cut7Δ* double mutant cells. Daughter pole disorganization additionally affects cytoplasmic astral microtubule arrays. Mitotic events can influence licensing and semi-conservative centrosome replication in the succeeding G1/S. In human cells, separase and polo kinase license centrosomes for duplication in the next cell cycle during mitosis^39^. Whether loss of kinesin-14 Pkl1 impacts subsequent cell cycle events outside of mitosis is not known. However, the changes to daughter spindle pole integrity without Pkl1 indicate a broader role beyond microtubule nucleation for spindle assembly, such as maturation or integrity of the daughter MTOC. We did not detect a similar role with kinesin-5 at the daughter MTOC and additional loss of Cut7 does not exacerbate these phenotypes. The concept of asymmetric events at spindle poles is well known. In budding yeast, γ-tubulin mutants have been isolated that block robust microtubule nucleation from a single pole as seen by transmission electron microscopy^40^. In human cells, mother centriole stability is asymmetrically affected by kinesin-13 Kif24 (ref. 41). As well, regulation of poles can be asymmetric and is observed in mitotic checkpoint pathways^26^,^34^,^35^,^42^,^43^,^44^,^45^,^46^,^47^ that monitor spindle assembly, positioning and timing to help ensure the accurate segregation of chromosomes in cell division.

In this study, we demonstrate that the role of kinesin-14 at γ-TuRC is to block microtubule nucleation and that key domains are required for this mechanism. This ability to localize to γ-TuRC at spindle pole bodies is conserved with γ-TuRC in the mammalian centrosome. We additionally identified the ability of kinesin-5 to bind γ-TuRC as a key component in the Klp/γ-TuRC regulatory mechanism in fission yeast. Pkl1 interacts with γ-TuRC through two domains, a Motor domain that binds to γ-tubulin helix 11 and distinct Tail domain binding to the complex. The combined domains provide strongest interactions with γ-TuRC^17^. Our data indicate that similar to Pkl1, the Tail domain of Cut7 is the primary γ-TuRC targeting element. We hypothesize that the Motor domain plays a role in assisted targeting to the γ-TuRC site at spindle poles and in competition with Pkl1 binding to this site. Consistent with our previously published findings on Pkl1, we observe that the combined domains of Cut7 provide the strongest interactions with γ-TuRC. However, unlike Pkl1 that has low affinity to microtubules^48^, Cut7 retains the ability to bind strongly to spindle tubulins when γ-tubulin specific binding is prevented. This alternative site of interaction may lower the pool of Cut7 at γ-TuRC. Thus, as a consequence of blocked loading due to the helix 11 mutation and retained high microtubule binding affinity, we would expect reduced binding of Cut7 to γ-TuRC complexes with γ-tubulin-K5A present as compared with Cut7ST, as observed. Interestingly, the *cut7-22* mutation lies within a MAP kinase phosphorylation consensus sequence in the conserved BimC sequence of the Cut7 Tail domain^29^, indicating that phosphorylation at this or other kinase sites within this domain may be important in the γ-TuRC mechanism. Finally the dual regulatory relationship of kinesin-14 and kinesin-5 at the γ-TuRC in fission yeast, along with the ability of PγTR peptide to block nucleation and spindle bipolarity in breast cancer cells, is expected to be impactful in regard to cancer therapy^49^. We are interested in exploring development of this new class of mitotic spindle protein (MSP) reagents as an addition to combined cancer therapies, in particular with Ispinesib/Monastrol^50^,^51^ that targets human kinesin-5 or in response to taxol-resistant cancers.

Our findings are of particular interest in regard to multiple clinically oriented studies^52^,^53^,^54^,^55^,^56^,^57^,^58^,^59^,^60^ that demonstrate overexpression of γ-tubulin and other centrosomal proteins is characteristic of tumorigenesis and human malignancies in multiple tissues. In this case supernumerary microtubule-nucleating centrosomes are often observed and result in abnormal multipolar mitoses, aneuploidy and ultimately cell death^58^,^59^,^61^. Similarly, overexpression of γ-tubulin in malignant cells can also produce ectopic microtubule nucleation in the cytoplasm. This is thought to result from γ-tubulin-centrosome decoupling as well as subcellular sorting changes to soluble cytoplasmic pools or insoluble centrosomal complexes^52^,^57^,^58^,^60^ as well as insoluble cytoplasmic aggregates^53^. Interestingly, an increase in the percentage of soluble cytoplasmic γ-tubulin is associated with cell lines of higher aggressiveness and poorer prognosis versus those of low or moderate aggressiveness^58^. Further, γ-tubulin can be incorporated within the α-/β-tubulin lattice of cytoplasmic microtubules that may impact drug resistance^58^. The ability of the PγTR peptide to target complexed γ-tubulin could allow a means to prevent ectopic microtubule nucleation, although currently this is untested. Regardless, the peptide can mitotically arrest both the MCF-7 low and MDA-MB-231 high aggressive cell lines with similar efficiency. The benefit of PγTR versus other anti-mitotic agents that solely target microtubules is yet to be determined but is of interest in particular for malignant cell lines that are difficult to arrest and that often develop resistance.

## Methods

### General yeast strains and growth conditions

Standard procedures for genetic manipulation of fission yeast are as described^62^ (*S. pombe* strains in Table 1). Cultures grown in fully supplemented YES-rich medium or minimally supplemented medium are also as described^62^. For yeast transformations, we used the EZ-YEAST Transformation Kit (MP Biochemicals). In growth assays, cells were grown to logarithmic phase in 10 ml rich YES media at 27 °C. Cells were counted by haemocytometer and equalized and spotted at an initial concentration of 2 × 10^7^ cells ml^−1^ with followed by 1/10 serial dilutions. Plates at 30 °C and 36 °C were grown for 4–5 days (*n*=3 experiments). Plates at 25 °C were grown for 7 days. For promoter induction using the pREP81 low strength^63^ or pREP90x high strength^64^ nmt plasmids, cells were maintained on plates containing 5 μg ml^−1^ thiamine before inoculation in 10 ml selective media with (control) or without (test) 5 μg ml^−1^ thiamine for 17 h. Plates used to assess viability contained 5 mg l^−1^ Phloxine B (Sigma-Aldrich). Mini chromosome loss was measured as described^65^. Growth curves were obtained using haemocytometer.

### Yeast strain constructions

Integration of the *ura4* gene at the *cut7* locus was done using a PCR-based gene-targeting approach with long tracts of flanking homology as previously described^66^ (Epicentre MasterAmp Extra-Long PCR Kit). We used 500 bp homology upstream and downstream of the *cut7* open reading frame and verified stable integrants by colony PCR (Fig. 1a). Plasmid Integration was done with pREP vectors using homologous recombination at the autonomous replication site (Mlu1, New England Biolabs). All genetic crosses were done on minimal sporulation media, followed by marker selection and colony PCR.

### Synchronous yeast culture

Cultures were grown overnight in YES-rich or selective minimal media at 27 °C using baffled flasks. Hydroxyurea (11 mM) was added to cultures in logarithmic phase and incubated for 4 h. Cells were then washed twice with 10 ml sterile water before release in fresh media. Depending on the experiment, cells were released at 27 °C (permissive temperature), 32 °C (microtubule repolymerization) or 36 °C (restrictive temperature).

### FPLC sedimentation and immunoprecipitate analysis

Yeast whole-cell extracts were prepared using mechanical bead beating (Mini-Beadbeater-16, Biospec) in Buffer P (50 mM Na_2_PO_4_ pH 7.2, 10% glycerol, 150 mM NaCl 5 mM ATP, 100 μM GTP])with protease inhibitors (PMSF-1 mM, Leupeptin-50 μM, Pepstatin-2 μM, Aproptinin-175 nM and Pefabloc-200 μM). Three centrifugations at 17,000 *g* (1 min, 5 min, 30 min) were used to clarify cell extracts. Separose 6 FPLC was performed as described^15^. For immunoprecipitation, whole-cell extracts were incubated with anti-V5-tag mAb-Magnetic beads (MBL International) at 4 °C for 30 min. Beads were washed three times with Buffer P before elution by boiling and immediate analysis by western blotting. Pkl1 peptide co-immunoprecipitation assays were performed as previously described^17^. Antibodies used were primary mouse anti-γ-tubulin monoclonal (1:10,000; Sigma-Aldrich cat. T5326), primary rabbit anti-HA epitope tag (1:5,000; Rockland cat. 600-401-384), primary rabbit anti-FLAG polyclonal (1:320; Sigma-Aldrich cat. F7425), primary mouse anti-V5 monoclonal (1:5,000; Life Technologies cat. R96025) or mouse anti-V5 IgG HRP conjugated monoclonal (1:5,000; Life Technologies cat. R96125), goat anti-rabbit IgG HRP conjugate (1:20,000; Millipore cat. 12-348) and goat anti-mouse IgG HRP conjugate (1:10,000; Novagen cat. 71045).

Human lysates were prepared by harvesting confluent cells with 2 ml TrypLE (Life Technologies) and centrifuging for 5 min at 1,000 r.p.m. followed by two washes with 1 ml 1X PBS. Cells were lysed by incubation on ice in RIPA+ Buffer (Tris–HCl pH 7.5, 50 mM, NaCl 150 mM, 1% Triton X-100, 1% deoxycholic acid sodium salt, 0.1% SDS; supplemented with Leupeptin 5 mM, Pepstatin 2 μM, Aprotinin 175 nM, PMSF 1 mM+GTP 100 μM) for 45 min, mixing occasionally. Lysates were clarified by centrifugation at 14,000 r.p.m. (20,817 *g*) at 4 °C for 1 h. Peptide co-immunoprecipitation assays were performed as above. Antibodies used for western blots were primary mouse anti-γ-tubulin monoclonal (1:9,000; Sigma-Aldrich cat. T5326) and primary rabbit anti-GCP2 polyclonal (1:2,000; Thermo Scientific cat. PIPA521433). The secondary antibodies mentioned above were used for detection by HRP. Uncropped scans of western blots are provided in Supplementary Figs 1 and 2.

### Breast cancer cell culture and peptide transfection

MCF-7 and MDA-MB-231 (ATCC) cells were maintained in 25 cm^2^ tissue culture treated Corning flasks (Sigma-Aldrich) in DMEM complete medium with Glutamax-1 and supplemented with 10% fetal bovine serum. MCF-7 DMEM complete medium was additionally supplemented with 0.01 mg ml^−1^ bovine insulin (Sigma-Aldrich). Flasks were maintained at 37 °C in 5% CO_2_, 95% air, and cells were passaged every 5–7 days using 1 ml TrypLE (Life Technologies).

For live cell peptide transfection we used the Chariot system (Active Motif) according to the manufacturer’s instructions. Cells were seeded into 35 mm tissue culture-treated dishes containing coverslips and grown in complete medium to ~60% confluency. One microgram of kinesin-14 Tail peptide PγTR (GenScript) was diluted in 100 μl 1 × PBS on ice. Six microlitres of 1/10 PBS-diluted Chariot reagent was further diluted into 100 μl sterile water on ice in a separate tube. Diluted peptide and diluted Chariot were combined and incubated at room temperature for 30 min to allow the Chariot-peptide complex to form. Following incubation, media was aspirated from cells, which were then washed once in 1 × PBS. The entire 200 μl volume was added to cells in 400 μl serum-free media and gently rocked to ensure even delivery. Plates were returned to 37 °C for 1 h. Next, DMEM complete growth medium was added to 2 ml without removing the peptide delivery solution (108 μM peptide in 2 ml). Cells were further incubated at 37 °C overnight and fixed after 24 h.

### Fluorescence microscopy and immunocytochemistry

Fluorescence microscopy was performed using Zeiss Observer.Z1 inverted microscope with 63X Plan-Apochromat 1.4 NA oil and × 100 oil 1.45 PlanFLUAR DIC objectives. Data were obtained using a Hamamatsu ORCA ER CCD camera with Zeiss Axiovision Rel 4.8 acquisition software. We acquired 20-image 0.1 μm Z-stacks. Timelapse series were acquired every 30 s to 6 min, with a median interval of 2 min. The 10-image 0.1-micron Z-stacks were superimposed on each timelapse image in a series. With live cells, GFP-Atb2 was imaged at 50–60 ms exposure and mCherry-Atb2 was imaged at 500 ms exposure. Only GFP-Atb2 was used in timelapse. Average temperature in the imaging room was 23 °C. Using −20 °C methanol fixation, we were able to preserve GFP signal for quantifying phenotypes. In immunocytochemistry, microtubules were stained with a primary TAT1 antibody (1:25)^67^, followed by secondary goat anti-mouse Alexa Fluor 488 IgG (1:50; Life Technologies cat. A-11001) and DNA was stained in 1 μg ml^−1^ Hoechst. A monoclonal anti-V5 primary antibody conjugated to FITC was used for viewing V5-tagged Cut7 constructs in fixed cells (1:500; Life Technologies cat. R963-25). Cells were imaged immediately using the Zeiss Observer.ZI system. In Fig. 8e, zoomed images were made high contrast. Z-stacks were made into 2D projections using ImageJ. Cold depolymerization and repolymerization of *in vivo* microtubules was performed as previously described^68^. Wild type and *pkl1Δ cut7Δ* cells containing the integrated *GFP-atb2* (α-tubulin) plasmid were fixed to preserve GFP signal and analysed using the Zeiss system.

Human cells were fixed on glass coverslips in −20 °C methanol for 10 min, washed with 1 × PBS and permeabilized in 0.5% Triton X-100 for 20 min. Following further washes, cells were blocked for 30 min in 1% bovine serum albumin/knock out serum replacement (BSA/KOSR). For peptide localization to centrosomes, 1 μg ml^−1^ of peptide was applied after blocking and before primary antibody application. Antibodies used were primary mouse anti-γ-tubulin monoclonal (1:5,000; Sigma-Aldrich cat. T5326) or primary mouse anti-α-tubulin monoclonal DM1A (1:1,000; Santa Cruz Biotech cat. sc-32293), primary His-tag polyclonal antibody (1:1,000; Cell Signaling cat. 2365), secondary goat anti-mouse Alexa Fluor 488 IgG (1:1,000; Life Technologies cat. A-11001) and secondary goat anti-rabbit Texas Red (1:1,000; Life Technologies cat. T-6391). Antibodies were diluted in 1% BSA/KOSR antibody dilution buffer. After secondary antibody application and washes, DNA was stained with 1 μg ml^−1^ Hoechst solution in 1 × PBS for 10 min followed by three times final PBS washes and mounted on slides with ProLong Gold anti-fade (Life Technologies). The 40-image 0.1 μm Z-stacks were made into maximum intensity 2D projections using ImageJ.

### *In vitro* microtubule nucleation assays

*In vitro* microtubule nucleation assays were performed in a total volume of 5 μl. That is, 3 μl for the sample and 2 μl of tubulin at a 1:5 ratio of Rhodamine:unlabelled tubulin (Cytoskeleton). Total tubulin concentration was 3.75 μg μl^−1^ in 2.5 × tubulin working buffer (2.5 × BRB80: 200 mM PIPES, 2.5 mM MgCl_2_, 2.5 mM EGTA at pH 6.8 and 2.5 mM GTP). For whole-cell extract nucleation analysis with peptide, 2 μl of whole-cell extract was added with 1 μl of peptide at 300 nM for a final peptide concentration of 60 nM. This 3 μl combination was added first followed by tubulin working buffer. For samples with no peptide, the 5 μl final volume comprised 3 μl RIPA buffer and 2 μl of whole-cell extract. The 5 μl reaction was combined on ice, quickly spun and returned to ice before incubating in a 37 °C water bath for 4 min. Sample incubation was staggered at 20 s intervals to allow for pipetting. After 4 min, 50 μl of 1% glutaraldehyde fixing solution was added and tubes were incubated at room temperature for 3 min. Samples were completed by addition of 1 ml 1 × BRB80, inverting multiple times to mix. For analysis, 50 μl of this mixture per sample was sedimented by ultracentrifugation at 173,000 × *g* through a 15% glycerol cushion onto glass coverslips and imaged by Rhodamine epifluorescence using the Zeiss system at × 630. Images of multiple fields were collected and the average microtubule number per field was determined.

### Structural analysis

PyMol molecular visualization software (V1.5) was used for structural analysis of the conserved α-β-tubulin heterodimer 1TUB^10^ and conserved γ-tubulin monomer 1Z5V^6^,^11^,^14^,^69^ in Fig. 3.

### Statistical analysis

For statistical analysis of phenotypes, *n* values were chosen as number of cells per strain needed to ensure adequate power to detect significant outcomes. *P*-values were generated using Student’s *t*-test and statistical significance was considered for *P*<0.05 as appropriate. All statistical data in this study are reported as mean±s.d. or ±s.e.m., as indicated. For cell cycle arrest by 1 μg transfection of PγTR, 12 fields at × 200 were counted. Arrested cells with positive peptide signal were taken as a percentage of the entire population. Cells that were negative for peptide signal did not arrest.

## Author contributions

J.L.P. and Z.T.O. designed experiments and wrote the manuscript. Z.T.O. performed genetics, strain construction, cell biology microscopy, cell culture and constructed figures. A.G.C. assisted in microscopy and performed fission yeast biochemistry. T.D.R. performed *in vitro* microtubule nucleation, human biochemistry, yeast tetrad dissection and serial growth assays.

## Additional information

**How to cite this article:** Olmsted, Z. T. *et al*. Kinesin-14 and kinesin-5 antagonistically regulate microtubule nucleation by γ-TuRC in yeast and human cells. *Nat. Commun.* 5:5339 doi: 10.1038/ncomms6339 (2014).

## Supplementary Material

Supplementary Information Supplementary Figures 1-2

## Figures and Tables

**Table 1:** *Schizosaccharomyces pombe* strains used in this study.

**Strain**	**Genotype**
FY392	*h- his3-D1 leu1-32 ade6-M210 ura4-D18*
JP163	*h+ his3-D1 leu1-32 ura4-D18 ade6-M216 pkl1D::his3+*
JP164	*h- his3-D1 leu1-32 ura4-D18 ade6-M216 pkl1D::his3+*
JPZO47	*h+ his3-D1 leu1-32 ura4-D18 ade6-M216 pkl1D::his3+ cut7D::ura4+*
JPZO48	*h- his3-D1 leu1-32 ura4-D18 ade6-M216 pkl1D::his3+ cut7D::ura4+*
JP181	*h+ his3-D1 leu1-32 ura4-D18 ade6-M216 cut7-22ts*
JP183	*h+ his3-D1 leu1-32 ura4-D18 ade6-M216 pkl1D::his3+ cut7-22ts*
JPZO49	*h- his3-D1 leu1-32 ade6-M210 ura4-D18 [ars1::prep41GFP/atb2]*
JPZO50	*h+ his3-D1 leu1-32 ura4-D18 ade6-M216 pkl1D::his3+ [ars1::prep41GFP/atb2]*
JPZO51	*h+ his3-D1 leu1-32 ura4-D18 ade6-M216 pkl1D::his3+ cut7D::ura4+ [ars1::prep41GFP/atb2]*
JPZO52	*h+ his3-D1 leu1-32 ura4-D18 ade6-M216 cut7-22ts [ars1::prep41GFP/atb2]*
JPZO53	*h+ his3-D1 leu1-32 ura4-D18 ade6-M216 pkl1D::his3+ cut7-22ts [ars1::prep41GFP/atb2]*
JPZO54	*h+ his3-D1 leu1-32 ura4-D18 ade6-M216 cut7-22ts [ars1::pJPGFP/gtb1]*
JPZO55	*h+ his3-D1 leu1-32 ura4-D18 ade6-M216 pkl1D::his3+ cut7-22ts [ars1::pJPGFP/gtb1]*
JP114	*h- his3-D1 leu1-32 ura4-D18 ade6-M210 pcp1-GFP::G418*^*R*^
JP265	*h- his3-D1 leu1-32 ura4-D18 pcp1-GFP::G418*^*R*^ *pkl1D::his3+*
JPZO56	*his3-D1 leu1-32 ura4-D18 pcp1-GFP-G418*^*R*^ *pkl1D::his3+ cut7D::ura4+*
JPZO57	*h- his3-D1 leu1-32 ura4-D18 pcp1-GFP::G418*^*R*^ *[ars1::prep81GFP/P450]*
JPZO58	*h- his3-D1 leu1-32 ura4-D18 pcp1-GFP::G418*^*R*^ *pkl1D::his3+ [ars1::prep81GFP/P450]*
JPZO59	*h+ his3-D1 leu1-32 ura4-D18 pcp1-GFP::G418*^*R*^ *pkl1D::his3+ cut7D::ura4+ [ars1::prep81GFP/P450]*
JPZO60	*h- his3-D1 leu1-32 ura4-D18 pcp1-GFP::G418*^*R*^ *[ars1::prep41mCherry/atb2]*
JPZO61	*h- his3-D1 leu1-32 ura4-D18 pcp1-GFP::G418*^*R*^ *pkl1D::his3+ [ars1::prep41mCherry/atb2]*
JPZO62	*his3-D1 leu1-32 ura4-D18 pcp1-GFP::G418*^*R*^ *pkl1D::his3+ cut7D::ura4+ [ars1::prep41mCherry/atb2]*
JPZO63	*h+ his3-D1 leu1-32 ura4-D18 ade6-M216 pcp1-GFP::G418*^*R*^ *pkl1D::his3+ cut7D::ura4+ [pREP81FLAG/pkl1]*
JPZO64	*h+ his3-D1 leu1-32 ura4-D18 ade6-M216 pkl1D::his3+ cut7D::ura4+ [pREP90x/pkl1D95]*
JP81	*h- leu1-32 ura4-D18 ade6-704 [pSp(cen1-7L)sup3E::ura4+]*
JPZO65	*leu1-32 ura4-D18 ade6-704 pkl1D::his3+ [pSp(cen1-7L)sup3E::ura4+]*
JPZO66	*leu1-32 ura4-D18 ade6-704 pkl1D::his3+ cut7D::ura4+ [pSp(cen1-7L)sup3E::ura4+]*
JP113	*h+ leu1-32 ura4-D18 ade6-M210 mad2D::ura4+*
JPZO67	*his3-D1 leu1-32 ura4-D18 pkl1D::his3+ cut7D::ura4+ mad2D::ura4+*
CoIP16	*h- leu1-32 mad2-eGFP::ura4+*
JP113	*leu1-32 pkl1D::his3+ mad2-eGFP::ura4+*
JPZO68	*leu1-32 pkl1D::his3+ cut7D::ura4+ mad2-eGFP::ura4+*
JPZO69	*leu1-32 pkl1D::his3+ cut7D::ura4+ mad2-eGFP::ura4+ [ars1::prep41mCherry/atb2]*
JP136	*h- leu1-32 ura4-D18 ade6-M210 gtb1-PL302*
JPZO70	*leu1-32 ura4-D18 ade6-M210 gtb1-PL302 pkl1D::his3+ cut7D::ura4+*
JPZO71	*leu1-32 ura4-D18 ade6-M210 gtb1-PL302 pkl1D::his3+ cut7D::ura4+ [ars1::prep41GFP/atb2]*
JP129	*his7-336/his7- leu1-32/leu1-32 ura4-D18/ura4-D18 ade6-M210/ade6-M216 gtb1+/gtb1D::his7+*
JPZO72	*his7- leu1-32 ura4-D18 gtb1D::his7+ [ars1::prep81GFP/gtb1-K5A]*
JPZO73	*his7- leu1-32 ura4-D18 gtb1D::his7+ [ars1::prep81GFP/gtb1-K5A] pkl1D::his3+ cut7D::ura4+*
JPZO74	*his7- leu1-32 ura4-D18 gtb1D::his7+ [ars1::prep81GFP/gtb1-K5A] cut7D::ura4+*
JPZO75	*his7- leu1-32 ura4-D18 gtb1D::his7+ [ars1::prep42/gtb1-K5A] [pREP81FLAG/pkl1]*
JPZO76	*his7- leu1-32 ura4-D18 gtb1D::his7+ [ars1::prep42/gtb1-K5A] [pREP81V5/cut7]*
JPZO77	*his7- leu1-32 ura4-D18 gtb1D::his7+ [ars1::prep42/gtb1-K5A] [pREP81V5/cut7-HS]*
JPZO78	*his7- leu1-32 ura4-D18 gtb1D::his7+ [ars1::prep42/gtb1-K5A] [pREP81V5/cut7-ST]*
JPZO79	*h+ his3-D1 leu1-32 ura4-D18 ade6-M216 pkl1D::his3+ [pREP81FLAG/pkl1]*
JPZO80	*h+ his3-D1 leu1-32 ura4-D18 ade6-M216 pkl1D::his3+ [pREP90x/pkl1D95NLS]*
JPZO81	*h+ his3-D1 leu1-32 ura4-D18 ade6-M216 pkl1D::his3+ cut7D::ura4+ [pREP81FLAG/pkl1]*
JPZO82	*h+ his3-D1 leu1-32 ura4-D18 ade6-M216 pkl1D::his3+ cut7D::ura4+ [pREP81GFP/pkl1]*
JPZO83	*h+ his3-D1 leu1-32 ura4-D18 ade6-M216 pcp1-GFP::G418*^*R*^ *pkl1D::his3+ cut7D::ura4+ [pREP81FLAG/pkl1]*
JPZO84	*h+ his3-D1 leu1-32 ura4-D18 ade6-M216 pkl1D::his3+ [pREP81V5/cut7]*
JPZO85	*h+ his3-D1 leu1-32 ura4-D18 ade6-M216 pkl1D::his3+ cut7D::ura4+ [pREP81V5/cut7]*
JPZO86	*h+ his3-D1 leu1-32 ura4-D18 ade6-M216 pkl1D::his3+ cut7D::ura4+ [pREP81V5/cut7HS]*
JPZO87	*h+ his3-D1 leu1-32 ura4-D18 ade6-M216 pkl1D::his3+ cut7D::ura4+ [pREP81V5/cut7ST]*
JPZO88	*h+ his3-D1 leu1-32 ura4-D18 ade6-M216 pkl1D::his3+ cut7D::ura4+ [pREP81V5NLS/cut7ST]*
JPZO89	*h+ his3-D1 leu1-32 ura4-D18 ade6-M216 pkl1D::his3+ cut7D::ura4+ [pREP81V5NLS/cut7ST*^22^]
JPZO90	*h+ his3-D1 leu1-32 ura4-D18 ade6-M216 pkl1D::his3+ cut7D::ura4+ [pREP81V5NLS/cut7T]*
JPZO91	*h+ his3-D1 leu1-32 ura4-D18 ade6-M216 pkl1D::his3+ cut7D::ura4+ [pREP81V5NLS/cut7T*^22^]
LV15	*h- leu1-32 alp4-HA::G418*^*r*^
JPZO92	*h- leu1-32 alp4-HA::G418*^*r*^ *[pREP81V5/cut7]*
JPZO93	*h- leu1-32 alp4-HA::G418*^*r*^ *[pREP81V5NLS/cut7ST]*
JP272	*h- his3-D1 leu1-32 ura4-D18 ade6-M210 klp9-GFP::ura4+*
JP270	*h- his3-D1 leu1-32 ura4-D18 ade6-M210 pkl1D::his3+ klp9-GFP::ura4+*
JPZO94	*his3-D1 leu1-32 ura4-D18 pkl1D::his3+ cut7D::ura4+ klp9-GFP::ura4+*
JPZO95	*his3-D1 leu1-32 ura4-D18 pkl1D::his3+ cut7D::ura4+ klp9-GFP::ura4+ [ars1::prep41mCherry/atb2]*
JPZO96	*h- his3-D1 leu1-32 ura4-D18 pkl1D::his3+ [pREP82GFP/klp9] [ars1::prep41mCherry/atb2]*
JPZO97	*his3-D1 leu1-32 ura4-D18 cdc7-GFP::ura4+ pkl1D::his3+ cut7D::ura4+ [ars1::prep41mCherry/atb2]*
JPZO98	*his3-D1 leu1-32 ura4-D18 cut7-22ts pcp1-GFP::G418*^*R*^ *[ars1::prep41mCherry/atb2]*
