# Supplementary material for: Kinesin-14 and kinesin-5 antagonistically regulate microtubule nucleation by γ-TuRC in yeast and human cells
Source: Nat Commun. 2014 Oct 28;5:5339. doi: 10.1038/ncomms6339 (PMC4220466; doi:10.1038/ncomms6339)
Supplement: Supplementary Information — Supplementary Figures 1-2 [file ncomms6339-s1.pdf]

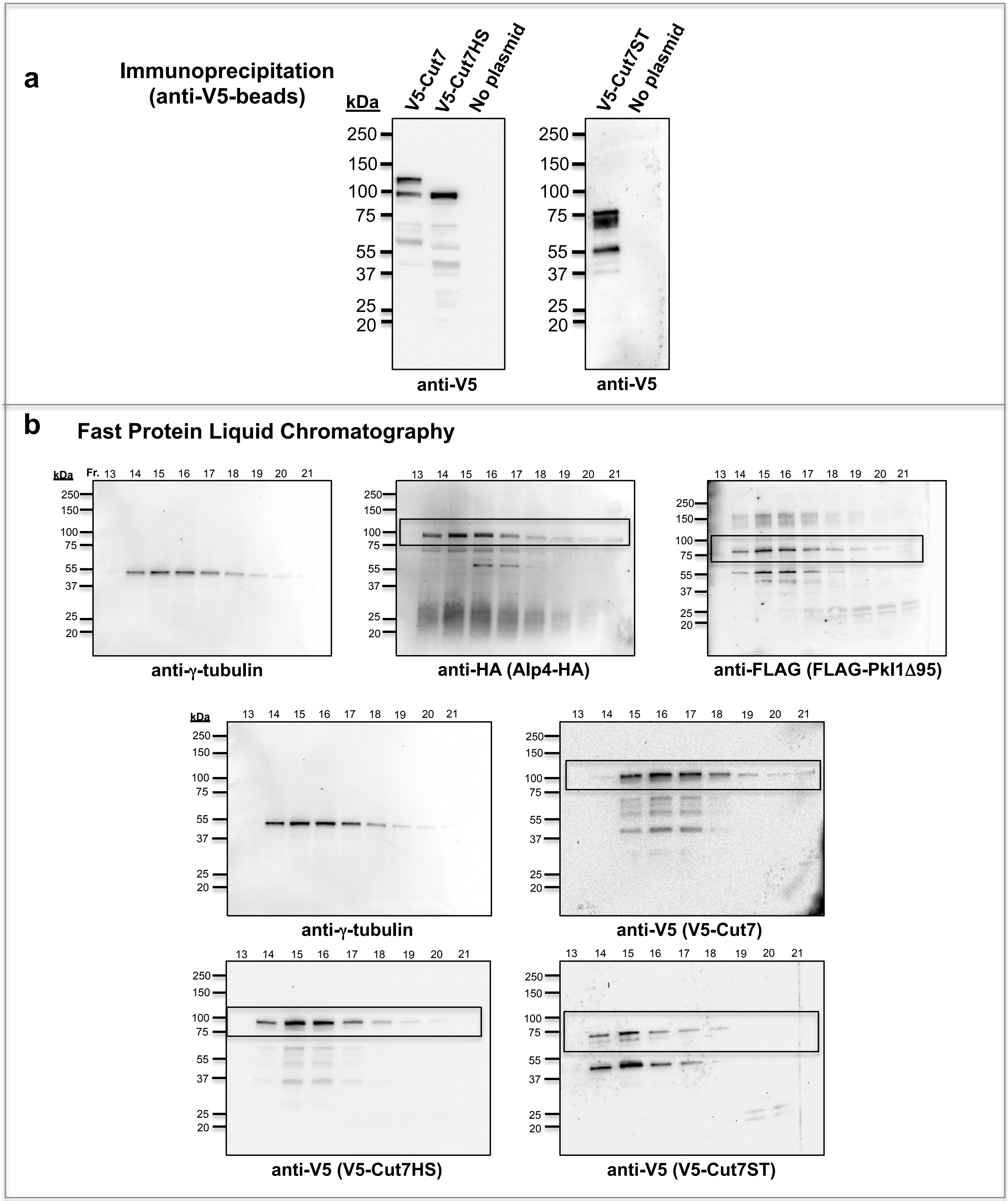

Supplementary Fig. 1

Uncropped Western blots from (a) immunoprecipitation and (b) FPLC assays from Fig. 2

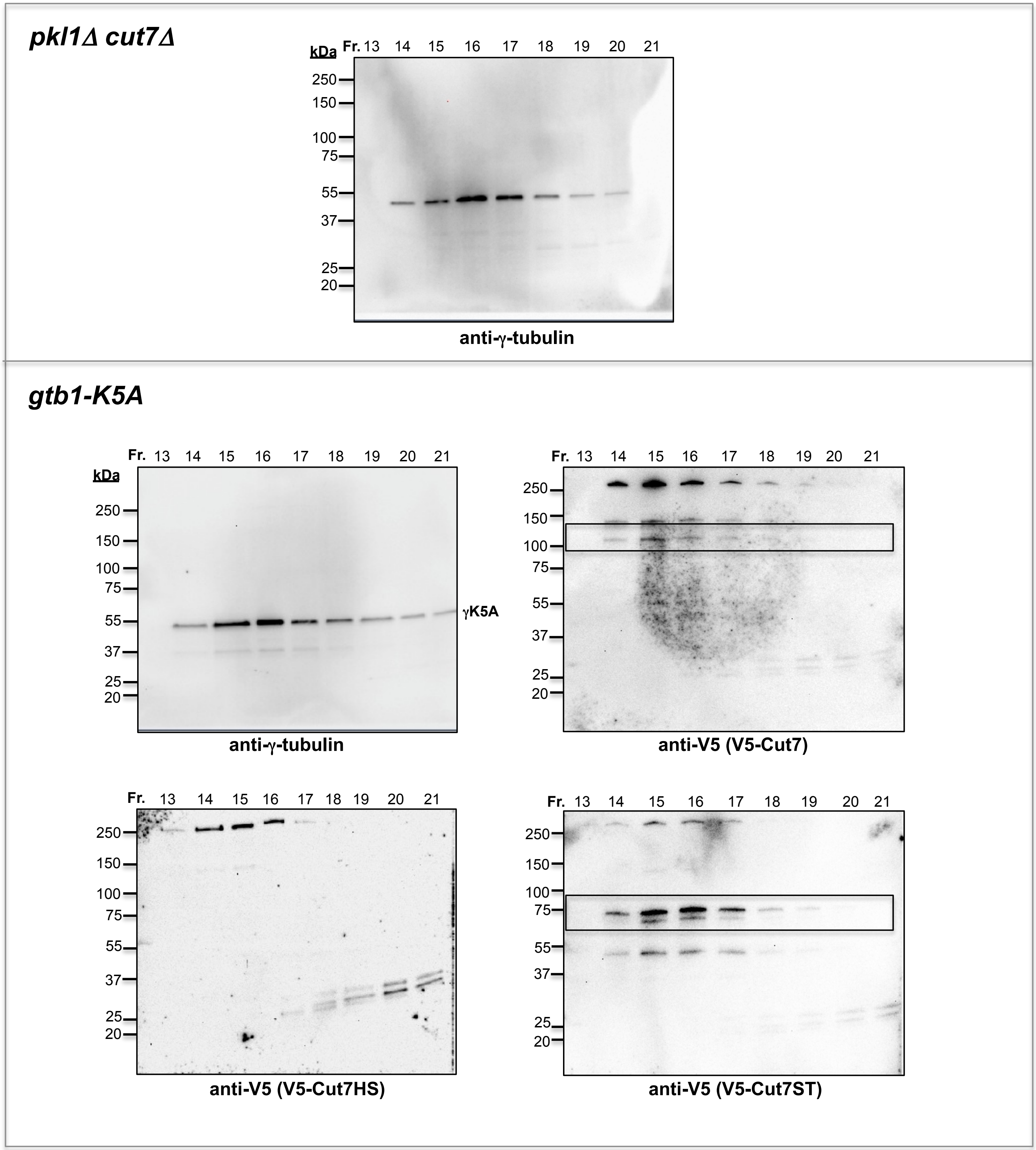

Supplementary Fig. 2

Uncropped FPLC Western blots from Fig. 3
